# Integrating Ecology and Economics in Agent‐Based Models to Support Agricultural Policy

**DOI:** 10.1002/advs.202520081

**Published:** 2026-10-26

**Authors:** Daniel Vedder, Judith Josephin Rakowski, Lea‐Deborah Kolb, Greta Sophie Theilen‐Loges, Sebastian Lakner, Guy Pe'er

**Affiliations:** ^1^ Department of Biodiversity and People Helmholtz Centre for Environmental Research – UFZ Leipzig Germany; ^2^ Institute of Biodiversity Ecology, and Evolution Friedrich‐Schiller‐Universität Jena Jena Germany; ^3^ German Centre for Integrative Biodiversity Research (iDiv) Halle‐Jena‐Leipzig Leipzig Germany; ^4^ Agro‐Ecological Modeling Group Institute of Crop Sciences and Resource Conservation University of Bonn Bonn Germany; ^5^ Department of Geographical Sciences University of Maryland College Park Maryland USA; ^6^ Faculty for Agricultural and Environmental Sciences University of Rostock Rostock Germany

**Keywords:** agent‐based modeling, agriculture, review, social‐ecological systems

## Abstract

Agriculture is a complex social‐ecological system with numerous interactions between policies, markets, farm management, landscapes, and ecosystems. Agent‐based models are a valuable method for studying these agricultural systems, as they are able to simulate the spatiotemporal dynamics and heterogeneous actors inherent to them. This potentially also makes such models helpful tools for scientific policy advice. Here, we conduct a literature review to evaluate how agent‐based models represent social‐ecological systems in agriculture. We analyze 56 published models using a set of 26 criteria to determine whether socio‐economic and ecological aspects of agriculture have been studied. We find that there is a particularly robust tradition of using agent‐based models for economic impact analyses of policy changes, and much effort has gone into studying decision‐making by land users. However, ecological models of biodiversity and ecosystem services are still often very abstract and ignore important land‐use processes, impeding their usefulness for providing policy advice. Research areas that are currently underdeveloped include simulating farm management and landscape dynamics in ecological models, risk management in economic models, and bidirectional human‐nature interactions in social‐ecological models. Agent‐based modeling of agriculture would also profit from using model coupling to represent more of the breadth of social‐ecological systems.

## Graphical abstract

Agent‐based models are valuable tools for studying social‐ecological systems in agriculture. This review maps how existing agent‐based models represent the human and natural domains of agriculture, and discusses developments in economic and ecological agent‐based modeling. It provides recommendations for moving toward a more integrated social‐ecological modeling of agricultural systems, in order to make agent‐based models more useful for policy support.

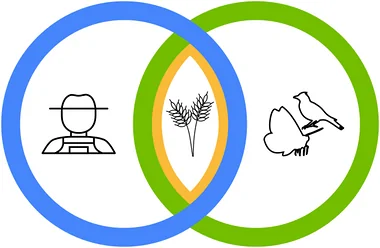

## 1 Introduction

Agriculture today faces a multitude of economic, social, and environmental challenges that urgently need to be addressed [1, 2]. In Europe, but also elsewhere, socio‐economic challenges include volatile markets, high regulatory burden, difficult working conditions, and rural depopulation [3]. These have contributed to a shrinking number of farms, an ageing rural population, and high farmer dissatisfaction [4, 5]. On the environmental side, modern agricultural practices have led to widespread pollution by agrochemicals, soil erosion and degradation, high greenhouse‐gas emissions, and ecosystem service losses [7]. They are also a leading contributor to biodiversity loss, with species in agricultural landscapes experiencing drastic declines across taxa [8, 9].

Worldwide there are numerous efforts to alleviate these problems, many of them based on policies [10]. One of the largest is the European Union's Common Agricultural Policy (CAP), a complex system of regulations and annual subsidies worth 55 billion Euros (as of 2026) [11]. Yet, even this has repeatedly failed to bring significant improvements [12, 13, 14]. On the contrary, the CAP has been criticized for primarily subsidizing large‐scale industrial agriculture, increasing the regulatory burden on farmers, and failing to set adequate environmental standards or incentives [15, 16]. Still, its continent‐wide scope, massive financial endowment, and global market impacts make it a key policy to effect environmental, social, and economic improvements in agriculture [17]. Other countries, too, are investing significant resources into their respective agricultural policies, and are facing similar hurdles [10].

The numerous interactions between the different aspects of agriculture mean that these challenges must be addressed in a concerted manner, using a social‐ecological systems (SES) perspective that considers both human and natural domains (Figure 1) [18, 19]. Otherwise, solution attempts may overlook the positive and negative interactions between the two, thus ignoring possible synergies and trade‐offs [20, 21] as well as feedback loops and tipping points [22, 23]. Furthermore, this close coupling of human and natural systems is as relevant for research as it is for policy. Accordingly, there is now a widespread agreement that studying SES necessitates a joint approach that crosses traditional disciplinary boundaries [24, 25].

**FIGURE 1:**
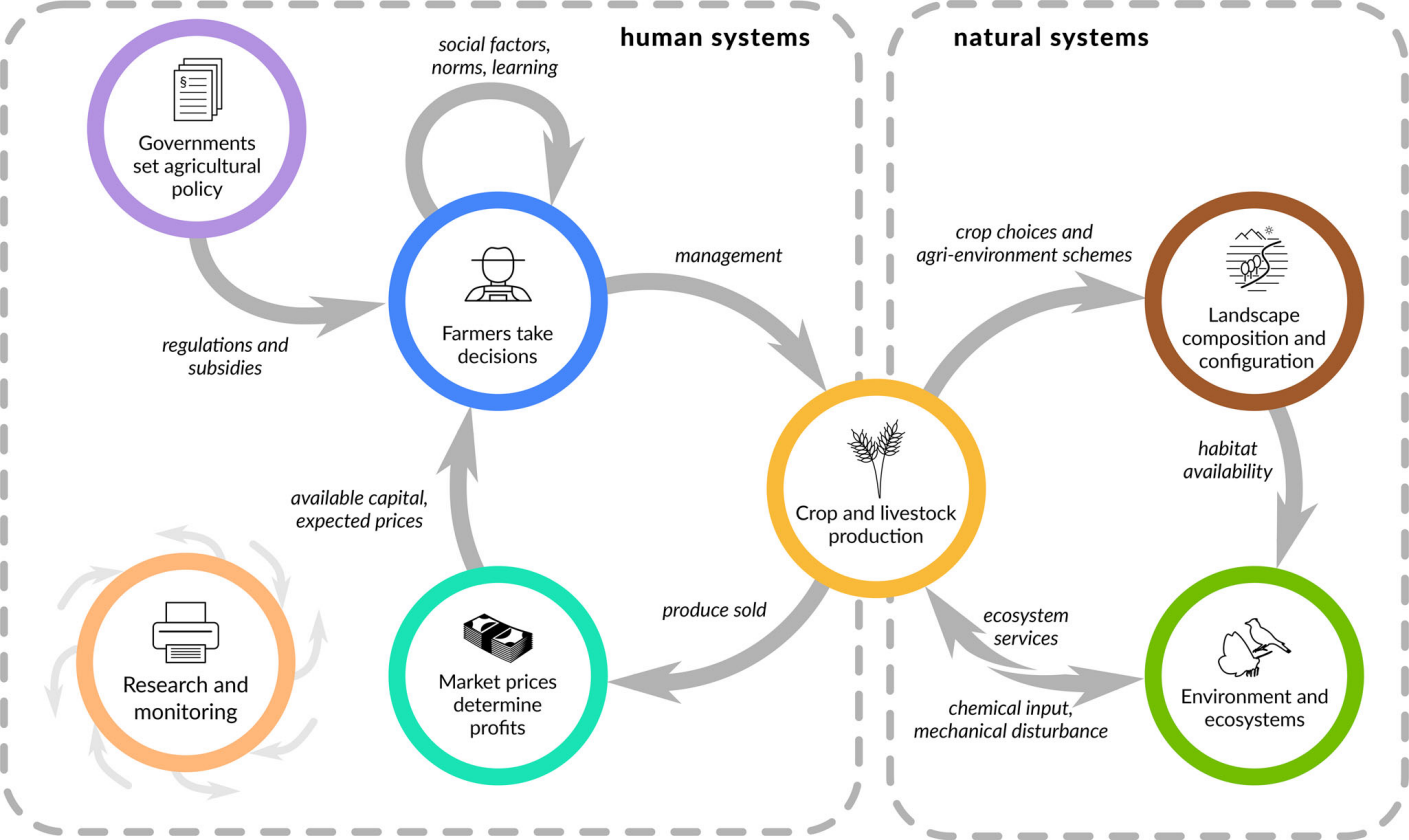
Agricultural systems contain multiple subsystems (circles) that have traditionally been perceived as categories of study for dedicated disciplines. Within a social‐ecological systems approach, the interrelationships (arrows) between the subsystems, and between the human and natural domains, are brought into focus. This allows a more comprehensive study of how changes to one part of the system may affect other subsystems, including the behavior of feedback loops and tipping points.

Agent‐based models (ABMs) are one important tool for studying social‐ecological systems. They are well‐established in multiple disciplines related to SES, including economics, political science, and ecology [26], as well as being used for integrated interdisciplinary analyses [27, 28]. They are a form of process‐based simulation model that represent complex systems as a collection of unique agents (such as farmers or animals), whose individual behavior and local interactions give rise to system‐level patterns [29]. This makes them conceptually well‐suited to simulating the heterogeneity and dynamic processes common to social and ecological systems, and analysing both spatial and temporal phenomena [30, 31, 32].

This suitability for SES research potentially also makes agent‐based models good tools to support decision‐makers in agricultural policy and management. Indeed, ABMs are frequently used for research on agricultural policy [33], including for policy impact assessments in the European Union [34, 35]. Still, their potential for policy support is not yet achieved, with impediments including issues such as lack of data availability or insufficient interaction between modelers and decision‐makers [36].

In this review, we evaluate to what extent current agent‐based models can be used to study social‐ecological systems and advise policy in an agricultural context. We focus on ABMs because of their conceptual suitability for SES research, and also because their widespread use in multiple relevant disciplines makes them an attractive method for interdisciplinary collaboration. Note that since the ecological literature usually refers to ABMs as individual‐based models, or IBMs, we keep this differentiation in the following, using “IBM” to denote specifically ecological models that have non‐human organisms as agents, while retaining the term “ABM” for agent‐based models more generally.

Specifically, we ask the following questions: First, how do ABMs from different disciplines represent the socio‐economic and ecological domains of agricultural SES (Figure 1), and where are possible areas of improvement? Second, to what extent do ABMs represent both domains, and how could the interactions between these be modeled better? And third, to what extent are ABMs already being used to evaluate policy, and how could their relevance for policy‐oriented SES research be increased?

Previous reviews in this area have focused on the general use of economic and/or environmental modeling for agricultural policy assessment [33, 34, 37], or the development of social‐ecological models more widely [38, 39]. Our work complements these by bringing together the perspectives of agricultural SES research, agent‐based modeling, and policy assessment, and provides an insight into the more recent work in this field. Our overall aim is to take stock of the state‐of‐the‐art of agricultural agent‐based modeling, and to develop recommendations for future disciplinary and interdisciplinary work on agricultural SES.

To achieve this aim, we conducted a systematic literature review to identify agent‐based models of agricultural systems. We evaluated whether elements of agricultural SES are represented in these models, using a catalogue of 26 criteria across 7 categories (Figure 2 and Table 1). This allowed us to describe the current state of the field, trace developments, and identify gaps.

**FIGURE 2:**
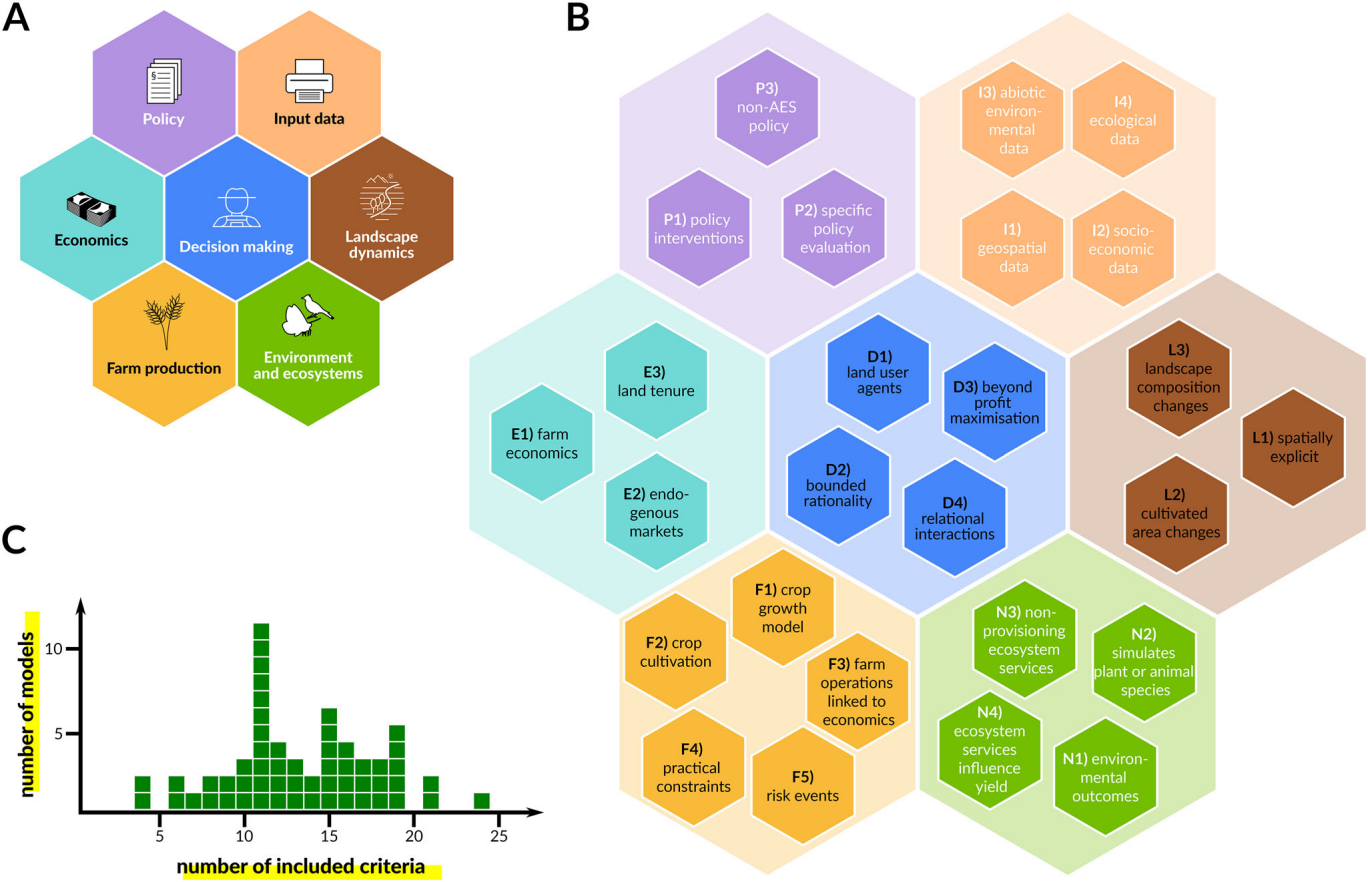
(A) Categories by which we assessed the comprehensiveness of models, based on the conceptual diagram in Figure 1. (B) Criteria that were checked within each category. Colors correspond to the categories in A, for criteria definitions, see Table 1. (C) Distribution of model comprehensiveness scores, showing how many models included how many criteria.

**TABLE 1:** Models found by the literature search were evaluated across seven categories of study, to reflect all major components of agricultural SES. We used 26 binary (yes‐or‐no) criteria to characterize how the models implemented each category.

Category	Criterion		Additional explanation
**Input data**	**I1**	The model uses geospatial data (e.g., remote sensing land cover maps, administrative field maps).	The model map is based on a real geographical area, rather than using an abstract, generated landscape.
	**I2**	The model uses empirical economic data (e.g., market prices, farm data).	e.g., from the EU's Farm Accountancy Data Network (FADN)
	**I3**	The model uses empirical data to parameterize abiotic environmental variables (e.g., weather, soil, nutrient flows).	
	**I4**	The model uses empirical data to parameterize ecological variables (e.g., species population sizes, green infrastructure, ecosystem service delivery).	
**Policy**	**P1**	The model assesses policy interventions, including hypothetical options.	Umbrella criterion (P2 and P3 necessarily include P1).
	**P2**	The model assesses real policy interventions, which have been or are planned to be implemented.	This includes *ex ante* or *ex post* assessments of policies such as new regulations of the CAP, or the Chinese “Grain‐to‐Green” programme.
	**P3**	The model assesses policy instruments *other than* area‐based payments for environmental measures (like the CAP's AECM or ecoschemes). This includes any other payments, directives, or market instruments of relevance to agriculture (e.g., direct payments, rural development funds, anti‐pollution regulations, trading agreements, labeling and certification, insurances).	
**Economics**	**E1**	The model considers farm economics (e.g., input and product prices, operating costs).	Umbrella criterion (E2 and E3 usually include E1).
	**E2**	The model uses endogenous input and/or output markets (i.e., uses demand and supply to calculate prices).	
	**E3**	The model considers land tenure (e.g., via land markets).	
**Decision‐making**	**D1**	The model has land users as decision‐making agents.	Umbrella criterion (D2, D3, and D4 usually include D1).
	**D2**	The model uses bounded rationality theory (i.e., farmers cannot perform absolute optimizations).	This may include satisficing, heuristic decision‐making, or optimization with a limited perception [63].
	**D3**	The model includes goals other than profit maximization (e.g., environmental stewardship, farming as tradition, risk aversion).	
	**D4**	The model considers relational interactions among farmers (e.g., imitation, cooperation, social norms).	
**Farm production**	**F1**	The model includes a crop‐growth model.	Crop growth and/or yield is calculated based on environmental and management parameters.
	**F2**	The model simulates crop cultivation (e.g., tillage, chemical input).	
	**F3**	Crop growth and/or farming operations are linked to farm costs and profits.	
	**F4**	The model considers practical constraints of farm operations (e.g., availability of labor force, driving distance).	
	**F5**	The model includes environmental and/or economic risk events.	Risks are expectable but unpredictable shocks that impact production and profits, e.g., market slumps or droughts. Models that include this criterion may or may not consider the response of land users to these risk factors (i.e., risk management).
**Landscape dynamics**	**L1**	The model is spatially explicit.	Umbrella criterion (L2 and L3 usually include L1).
	**L2**	The amount of land under active cultivation changes over time (e.g., land clearing, land abandonment, crop rotation with fallows).	
	**L3**	The landscape composition and/or configuration changes over time due to internal model processes (e.g., crop rotation, agri‐environment schemes).	
**Environment and ecosystems**	**N1**	The model includes abiotic and/or biotic environmental outcomes.	Umbrella criterion (N2, N3, and N4 usually include N1).
	**N2**	The model simulates individuals and/or populations of non‐domestic animals and/or plants.	This can include IBMs, (meta‐)population models, or analytic/statistical biodiversity models based on landscape structure.
	**N3**	The model considers non‐provisioning ecosystem service delivery as an output (i.e., regulating, supporting, or cultural services).	e.g., pollination, pest control, prevention of soil erosion, water retention and filtration, landscape aesthetics
	**N4**	The model considers feedback from regulating and supporting ecosystem services to production.	e.g., through coupling crop yield to pollinator abundance

## 2 Overview of Reviewed Models

Our main literature search yielded 505 papers, of which we classified 169 as relevant and selected 100 for further analysis, which amounted to 56 models (Table 2). An additional 12 papers related to these models were found through citations in the selected papers. Date of first publication of the models in our sample ranged between 2003 and 2025, with a median of three models published per year (range: 0–7). The reviewed models and their included criteria are depicted in Figure 3, while Figure 4 shows how many models included each criterion.

**TABLE 2:** List of analyzed models and their key references, sorted by the number of included criteria (most to least). Models that were not given a name by their authors are listed here under their first author's name. Only selected sources are given for models with many publications. The spatial scale is given qualitatively: continent > country > region > landscape > field. In the environmental outcome column, GHG = green house gas emissions and ESS = ecosystem services. The purpose is classified according to Edmonds et al. [110].

Model name	References	Region	Spatial scale	Spatial resolution	Temporal resolution	Environmental outcome	Purpose
MPMAS	[40, 41, 42, 44]	multiple	region	Field	daily	hydrology, soil, nutrients, GHG	explanation
ALMaSS	[48, 49, 113, 114]	Europe	landscape	1 m^2^	daily	biodiversity	prediction
SEEMS	[93, 115]	Asia	landscape	Field	annual	biodiversity, GHG	prediction
AgriPoliS	[50, 116, 117]	Europe	region	Field	annual	biodiversity, ESS, nutrients, hydrology	prediction
Aporia	[78, 118]	Europe	landscape	Field	annual	biodiversity, ESS	explanation
LUDAS	[119, 120]	Asia	landscape	not specified	annual	land use	prediction
SEALM	[121]	Africa	landscape	100 m^2^	annual	land use	explanation
SWISSLAND	[51, 122, 123, 124]	Europe	country	farm	annual	nutrients, pesticides	prediction
ALUAM‐AB	[53, 125]	Europe	landscape	1 ha	annual	land use	prediction
Bazzana et al.	[126]	Africa	landscape	field	annual	none	theoretical exploration
Roeder et al.	[127]	Europe	landscape	not specified	not specified	biodiversity	prediction
AgriLOVE	[54]	abstract	landscape	field	arbitrary	land use	theoretical exploration
PALM	[128, 129, 130]	multiple	region	n/a	annual	GHG	explanation
WICM	[68]	N America	landscape	1 ha	annual	Biodiversity, hydrology	explanation
‍AGRODEVS	[131, 132]	S America	Region	farm	annual	soil, pesticides, hydrology	prediction
CRAFTY	[22, 52, 73, 103]	Europe	continent	various	annual	biodiversity, ESS	explanation
‍Moretti & Bezanquen	[133]	Europe	country	n/a	annual	biodiversity	prediction
Schulze et al.	[27]	Europe	region	25 ha	annual	biodiversity, ESS	explanation
Delmotte et al.	[134]	Europe	region	field	annual	none	social learning
Granco et al.	[135]	N America	region	not specified	annual	hydrology, biodiversity	illustration
Pampas	[71, 136, 137]	S America	region	25 ha	annual	hydrology, land use	explanation
REGMAS	[138]	Europe	region	25 ha	annual	none	prediction
RF‐MAS	[139, 140, 141]	Australasia	region	not specified	annual	nutrients, GHG	explanation
SERA	[142, 143]	Europe	region	not specified	annual	biodiversity	explanation
ABM+LCA	[144, 145, 146, 147]	Europe	region	Field	monthly	GHG, nutrients	explanation
FARMIND	[65, 148, 149, 150]	Europe	region	n/a	annual	pesticides, nutrients, GHG	prediction
EFForTS‐ABM	[151, 152]	Asia	landscape	0.25 ha	annual	biodiversity, ESS	explanation
Martinet & Roques	[94]	abstract	landscape	1 ha	annual	ESS	theoretical exploration
Will et al.	[153]	Europe	region	field	annual	biodiversity	theoretical exploration
Bourceret et al.	[154]	abstract	landscape	arbitrary	annual	hydrology, nutrients	theoretical exploration
‍Le et al.	[155]	Asia	landscape	field	annual	land use	explanation
Tieskens et al.	[156]	Europe	landscape	1 ha	annual	none	social learning
Valbuena et al.	[157, 158, 159]	Europe	region	1 ha	annual	land use	explanation
AG‐ADAPT	[160]	Asia	region	1080 m	annual	none	explanation
‍AGRISP	[161]	Europe	region	field	annual	GHG, hydrology	prediction
ALABAMA	[162]	abstract	landscape	1 ha	not specified	biodiversity, water	theoretical exploration
AMBAWA	[163]	Africa	landscape	1 ha	half‐daily	ESS	prediction
Choquette‐Levy et al.	[58]	Asia	region	n/a	semi‐annual	none	explanation
Cong et al.	[75, 164, 165]	abstract	landscape	field	annual	ESS	theoretical exploration
‍de Jager et al.	[79, 166, 167]	Europe	region	1 ha	hourly	biodiversity	theoretical exploration
Drechsler	[168]	abstract	landscape	field	not specified	biodiversity	theoretical exploration
Gielda‐Pinas et al.	[169, 170]	Europe	landscape	25 ha	annual	nutrients, soil, water, land use	explanation
IFM‐CAP	[171, 172]	Europe	continent	n/a	not specified	none	prediction
SPASIMv1	[173]	Europe	landscape	0.1 ha	quarterly	wildfire risk	explanation
BEEHAVE	[174, 175]	Europe	landscape	not specified	daily	biodiversity	prediction
DYPAL	[176, 177]	Europe	landscape	7 m	annual	land use	explanation
Manson et al.	[178]	N America	region	25ha	annual	land use	description
GMSE	[69, 179]	abstract	landscape	arbitrary	arbitrary	biodiversity	theoretical exploration
FEARLUS‐SPOMM	[76, 180]	abstract	landscape	field	annual	biodiversity	theoretical exploration
EEEworm	[181, 182, 183]	abstract	3 m	1 cm^2^	hourly	biodiversity	explanation
TrophicLink	[77, 184, 185]	abstract	100 m^2^	not specified	daily	biodiversity	theoretical exploration
Bakam et al.	[186]	Europe	country	n/a	annual	GHG	prediction
Evans et al.	[187]	abstract	1 km^2^	5 m^2^	second	biodiversity	prediction
GRASSMIND	[188, 189, 190]	multiple	field	1 m^2^	daily	biodiversity	explanation
Meli et al.	[191, 192]	abstract	1 m^2^	1 cm^2^	hourly	biodiversity	explanation
Rands & Whitney	[193, 194]	abstract	landscape	field	n/a	ESS	theoretical exploration

**FIGURE 3:**
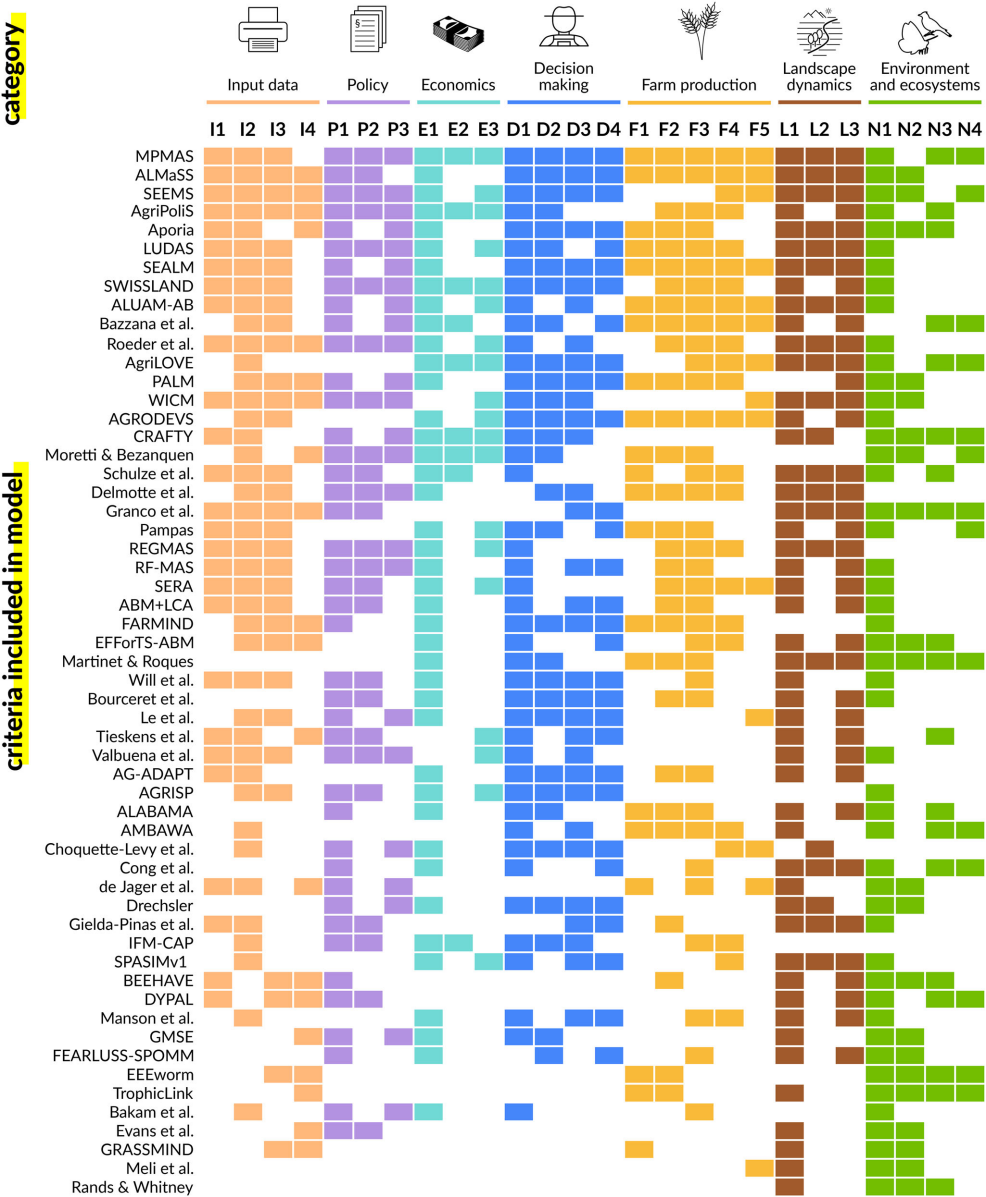
Table of criteria (columns) included in each model (rows). A colored tile denotes that this model includes this criterion. For criteria definitions, see Table 1.

**FIGURE 4:**
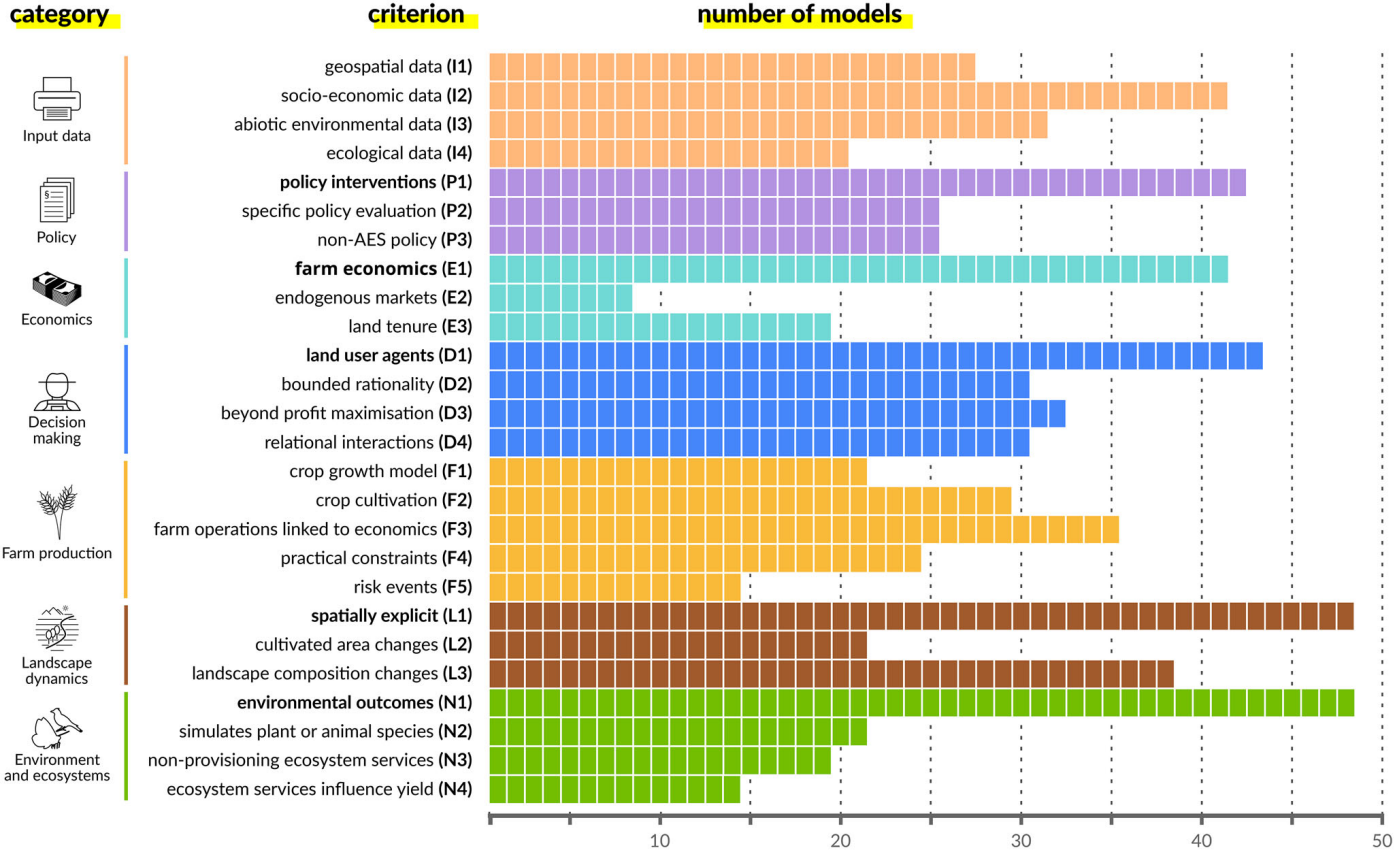
Number of models including each criterion (total number of models = 56). Each colored tile represents one model. Umbrella criteria are denoted by bold font. For more detailed definitions of the criteria, see Table 1.

The median number of criteria included by each model was 13, with a range of 4–24 (Figure 2c). There was no visible trend in the total number of criteria included by models over time, i.e., no visible increase or decrease in model complexity. For a more concrete (qualitative) insight into the state‐of‐the‐art in agricultural agent‐based modeling, we present two of the most comprehensive models in more detail (MPMAS and ALMaSS; Box 1 & 2).

Almost all models were spatially explicit and simulated environmental outcomes (L1 and N1, 86% each). More than two‐thirds of all models included the criteria “land user agent” (D1, 77%), “policy interventions” (P1, 75%), “farm economics” (E1, 73%), “socio‐economic data” (I2, 73%), and “landscape composition change” (L3, 68%). The rarest criteria were “endogenous markets” (E2, 14%), “risk events” (F5, 25%), and “ecosystem services influence yields” (N4, 25%). There was a weak trend toward including an increasing number of decision‐making criteria (D2, D3, D4) in newer models.

**Box 1: MPMAS**

MPMAS (Mathematical Programming‐based Multi‐Agent System) is an agricultural systems model that shares a common origin with AgriPoliS, another widely used model in our review. However, whereas AgriPoliS is explicitly an economic model (though it has been coupled to environmental modules), MPMAS was designed from the beginning to simulate the interactions between economic, social, and environmental processes [40].

It does so by simulating farmer households on a gridded landscape. Households are microeconomic units with individual properties (e.g., land endowment, labor supply, farm equipment, attitude to innovations) that maximize expected household income while considering additional goals. The resulting land use feeds into various biophysical submodels, which calculate crop yield, water flows, and soil quality. Different submodels are available for these tasks (both inbuilt and via model coupling), that can be selected based on the desired level of detail. The biophysical state of the landscape then feeds back into agents’ decision making. Within this general framework, several socio‐technical processes can be simulated, including irrigation, technology diffusion and land and water markets.

Although the main application focus of MPMAS is smallholder agriculture in developing countries, it has been applied to a broad range of case studies from across the world, including in Germany [41], Brazil [42], Uganda, and Chile [43]. Research questions include, for example, the evaluation of policies aimed at greenhouse gas reduction [42], competition between subsidies for bioenergy and biodiversity [41], and the adaptation of farmers to climate change [44].

**Box 2: ALMaSS**

ALMaSS is the most detailed biodiversity model in our review, and one of the three most comprehensive models overall. First published by Topping et al. (2003), it contains a farm module, a landscape module, and a variety of animal modules.

The farm module simulates the production of numerous crops, considering their seasonality, crop rotations, and cultivation actions such as ploughing and harvesting. Different farm types are initialized that use different crops and follow different management plans.

The landscape module simulates a real landscape, the weather (from historical weather data), and plant growth. Plant growth is modeled for each grid cell using species‐specific mathematical models dependent on the weather, season, fertilizer, or pesticide application, and cutting/harvesting. The model works at a 1 m^2^ spatial resolution and uses daily time steps.

Animal modules exist for over a dozen non‐domestic species from different taxa (including sky larks, roe deer, ground beetles, spiders, and voles), though only one species is simulated at a time. All animal modules are individual‐based and use a state/transition principle, i.e., individuals exhibit certain behavior (are in a certain state) until internal or external conditions cause them to transition to another state. Behavioral states typically include movement, territoriality, feeding, mating, and growth.

ALMaSS has been used to study species responses to pesticides [45], organic farming [46], and the Common Agricultural Policy [47, 48]. The farm module has been expanded to simulate more nuanced farmer decision‐making [49].

## 3 Lessons for Socio‐Economic Modeling

There is a robust tradition of using economic farm models in agricultural policy assessments. A particularly influential model in this tradition is AgriPoliS [50], but there are several other highly elaborated economic agent‐based models, such as MPMAS, SWISSland [51], and CRAFTY [52].

The biggest gap we see is the low representation of economic and environmental risks, particularly through external shocks (F5). Most ABMs that do include risk events mainly cover the effects of climate change [44, 53, 54]. However, risks (and the associated necessity of risk management) are an important area of study with regard to agricultural SES, for two reasons. First, the combination of climate change and environmental degradation increases the frequency and severity of fluctuations and shocks, both locally and globally, to which farmers have to respond [55]. Second, the reduction of risk through greater yield stability is an important argument for more environmentally‐friendly diversified farming systems, though one which remains poorly explored [56]. While there are other types of economic models and analysis techniques that deal with risks [57], agent‐based models could capitalize on their multidimensional representation of land users’ decision‐making to explore this topic from different perspectives. Therefore, given the importance of risk and risk management for social‐ecological transformations in agriculture [58], this is an important area for future ABMs to expand on.

An area that has seen a lot of work is the study of decision‐making processes by land users, especially farmers (D2, D3, D4). An increasing number of economics studies are going beyond the classical profit‐maximizing, fully‐rational agent (*homo oeconomicus*) to better understand the complexities of human decision‐making [59, 60]. This is also reflected in the agent‐based modeling literature of the last years [35, 61, 62]. Our review reveals widespread consideration of complex decision‐making processes, as well as the interconnections among behavioral and economic aspects of agricultural SES. Different nuances and components of decision‐making, including relationships and social networks, learning, and farmers’ backgrounds, are taken into account by three‐quarters of the models in our review [63]. Comparing this to the findings of prior reviews, it seems that there has been significant recent progress in the modeling of farmer decision‐making [33, 35, 38].

Still, we see several directions in which this can profitably be improved. The first is a more detailed study comparing the relative importance of different factors of decision‐making under different conditions [64]. Some such studies have already been carried out, for example using FARMIND [65] or ALMaSS [49]. As including more decision factors in a model also raises its susceptibility to error, further research into the dynamics of decision‐making should help social‐ecological modelers achieve a suitable level of complexity [62]. The second direction is to look at whether and how environmental processes feed back into farmer's decision‐making, e.g., through adaptation to increasing droughts or in response to pest cycles [66]. This would help to better recognize and explore the intricate interconnections between social and ecological processes in agriculture in a way that is so far only done very rarely [19, 67]. The third direction is to look at decision‐makers who are not themselves land users, such as consumers or policy‐makers. Again, a few models already do so, for example by including public utility companies [68] or conservation managers [69] as agents. However, most models focus exclusively on land users (typically farmers or farming households), thereby neglecting the wider societal context in which these persons operate. Expanding this, for instance to study different forms of governance systems [70], would be one way to incorporate more of the social breadth of SES into agent‐based models.

The role of markets has been modeled to very different degrees. While land markets are well‐represented in the existing socio‐economic ABMs (E3), at least partly due to the influence of AgriPoliS and MPMAS, markets for agricultural goods and products are rarely explicitly modeled (E2). Land markets play an important role in mediating structural change in agriculture, as farms grow or shrink in size or cease operation altogether. ABMs play to their strengths here, representing the individual decisions of many different farmers to study a large‐scale phenomenon [51, 71]. Endogenous markets for other goods and services are much rarer in our reviewed models. However, expanding models to also simulate markets for agricultural inputs and outputs could enable larger‐scale studies of entire food systems [72], and the exploration of sustainability pathways [73]. One way of accomplishing this may be by coupling ABMs to other economic models such as partial equilibrium models, which simulate market and price developments (as suggested by [37] and [35]).

## 4 Lessons for Ecological Modeling

About 60% of the models in our review considered biodiversity and/or ecosystem services in some form or another (N2, N3, N4). This reflects the importance of biodiversity conservation and ecosystem health in agricultural systems research. However, we observe several issues that negatively impact the ability of these models to help us understand agroecosystems and their utility for agricultural policy analyses.

First, and most importantly, many of the models are highly simplified with regard to ecology, either ignoring ecological processes or simulating very abstract systems. Several models use correlations with environmental values or indices based on landscape structure as a proxy for biodiversity or ecosystem services [74, 75]. Quite a few others do use process‐based models such as IBMs, but are very abstract and conceptual [76, 77]. Indeed, the lower part of Figure 3 shows a cluster of ecological models that are based on very little empirical data and include few other aspects of agricultural systems. This means that there are comparatively few ABMs or IBMs that can be used for applied studies of the impacts of agricultural policy and practice on the ecological processes of real landscapes and species [68, 78, 79]. We found this predominance of conceptual models surprising, as many IBMs in other contexts (such as forestry and fisheries) are often quite detailed and tend to be highly specific to contexts and species [80, 81]. This raises the question of why there are not more applied IBMs for agroecosystems? Given the success of applied IBMs in other ecosystems, this strikes us as a remarkably underexplored area of ecological research.

Second, many ecological models ignore temporal landscape dynamics and crop growth/cultivation. Whereas over 80% of the socio‐economic ABMs are spatially dynamic, only two‐thirds of the reviewed ecological IBMs simulate landscapes that change over time (L2, L3). This is despite extensive empirical research showing that spatio‐temporal dynamics of landscapes are among the most important drivers of biodiversity change in agroecosystems [82, 83]. Furthermore, only half of the ecological models also include crop growth and/or crop cultivation (F1, F2). Yet this would be important for three reasons. First, because the above‐mentioned landscape dynamics are the product of agricultural management. Second, because growth of crops and cultivation practices such as tillage or the application of agrochemicals play a major role in shaping biodiversity patterns [84]. And third, because a comprehensive ecological policy evaluation should be able to take into account changes in field‐level yields (both gains and losses) associated with biodiversity‐improving measures.

Finally, modeling the feedback from biodiversity and ecosystems to yields and farm economics remains a big challenge. Only a quarter of the analyzed models simulated ecosystem service effects on yield, making this one of the rarest criteria in our analysis. This is understandable, as quantitatively predicting levels of ecosystem service provision is notoriously hard and remains an active research question [85, 86]. However, being able to link crop growth models with landscape‐scale biodiversity models could be a decisive step toward a better understanding of biodiversity‐yield relationships [87], and would be a major step forward in making ecological models attractive and useful for policy makers and practitioners.

## 5 Implications for SES Modeling

With our paper we want to highlight the importance of large social‐ecological agent‐based models, by which we mean ABMs that include a broad range of categories and work with different types of empirical input data. This does not mean that every ABM needs to be large in this sense. The question of adequate model complexity has been discussed extensively before [88, 89, 90, 91]. There are many research questions to which smaller models (i.e., that include fewer categories or are more conceptual in nature) are better suited than a large model would be. Still, there are several open areas of research that require such larger models.

First, we observe that the critical influence of feedback loops on system resilience and tipping points has been accepted by SES researchers but is still rarely implemented in agent‐based modeling [92]. Rather than just studying uni‐directional effects of socio‐economic processes on ecological systems (or vice versa), we need models that can explore bi‐ or multidirectional interactions [93, 94]. Other authors have called for a better integration of climate change, land use, and biodiversity models [32, 95, 96]; this needs to be applied to agriculture, too. We posit that this will entail paying more detailed attention to crop and livestock production, as this is the nexus point linking farm management and its related socio‐economic processes to the environmental processes in the natural world (Figure 1).

Second, agent‐based modelers can only provide useful policy advice if their models can provide a reasonably realistic representation of the system and the policy in question [33, 89]. It is not surprising, that most of the reviewed models that evaluated specific policies (P2) also included much empirical input data (I1‐I4). Social‐ecological systems research has a valuable contribution to make to the study of agricultural systems [19, 20], but social‐ecological ABMs are still not well utilized in policy‐making [28]. One part of the problem is the lack of exchange between modelers and decision‐makers [35, 36]. Another challenge is the (perceived or actual) low reliability of ABM results, which requires rigorous validation of models intended to be used in policy [38, 97]. In this context, it is also critical to discuss and evaluate the uncertainty accompanying model design, parameterization, and output, in order to enable stakeholders to take informed decisions [98, 99]. Lastly, it is regrettable that although biodiversity conservation has long been a stated goal of agricultural policies such as the CAP, this is not yet reflected in the available models: in our review, only six out of 25 models that evaluated specific policies also simulated species [100].

We do recognize that building such large ABMs is not easy. One big difficulty lies in the disciplinary differences between the different modeling traditions. Although this has long been recognized [101], it continues to be a challenge [28]. One practical aspect of this is a frequent mismatch in the spatial and temporal scales of analysis [39]. For instance, it is notable that a clear majority of models in our review use annual time steps (Table 2), a temporal resolution that makes sense from an economics perspective but that is much too coarse for many ecological processes. A second aspect lies in the need to develop and use measurable indicators that are relevant and meaningful to both disciplines [14]. Finally, on a deeper level, the societal value debates surrounding land use (“environmental protection versus economic productivity”) can spill over into academic discourses and impede interdisciplinary collaboration.

The second big difficulty in building large ABMs comes from the technical challenges of building large software. Here, it can be instructive to consider the examples of MPMAS and ALMaSS, as the two most comprehensive models in our review (Box 1 & 2). Both were designed from the beginning to take a broad systems approach to studying agriculture, even though their background and main focus was recognisably economics and ecology, respectively. In implementing their conceptual design, they chose different technical approaches. ALMaSS is a very large, single code base, containing all features that have been added over the 20+ years of its use. This gives the advantage of all parts of the model integrating seamlessly with each other, at the cost of a very high technical complexity of the software. MPMAS on the other hand worked with model coupling from the beginning, implementing a small economic core model and linking this to a suite of already available biophysical models. This gives it flexibility to switch between different biophysical models for different research questions and reduces the programming work for the core development team, but means that the main model has to work within the constraints and limitations of the coupled models.

While both approaches can work well, we encourage social‐ecological modelers to work more with model coupling, as this is one of the quickest routes to creating truly social‐ecological models [102]. Several studies already do so, coupling multiple existing models in order to study the interlinkages between processes from different domains [72, 76, 103]. This technique has been used to great effect in other modeling disciplines, such as climate modeling [104], and is thus a promising avenue to pursue in future. (For one noteworthy example in land use modeling, see [102].) However, doing so will require a greater degree of code sharing among modelers [105], enabled by an increased use of open‐source repositories and standardized documentation [106, 107]. It also requires a thorough understanding of the modeling issues to consider [108], and a knowledge of appropriate software engineering practices [109].

## 6 Limitations

We recognize that in this study we have only been able to review a portion of social‐ecological models related to agriculture, specifically sampling agent‐based models that are relevant to agroecosystems. We also acknowledge that our criteria definitions (Table 1) leave some room for interpretation, and leave out other factors that can also be relevant to agricultural SES (e.g., technological advance or governance structures). Despite these caveats, we believe our literature review does offer a representative overview of the current state of agricultural agent‐based modeling, and illuminates recent trends and topics in the field. Still, we encourage our readers to look beyond our brief summaries and read the original publications to better understand how individual models work (Table 2).

On a more fundamental level, we want to address two scientific concerns related to the development of large, integrated ABMs. First, we want to reiterate our previous statement that large models are not, by mere virtue of their increased complexity, better than small models. Every model serves a specific purpose, with different purposes imposing different requirements and constraints on the developers [110]. Thus, model quality must be judged by adequacy for purpose, and not by comprehensiveness [91]. Therefore, while we concur with other authors that the study of SES will require some integrated models (and as we argue here, more than we currently have), we do not want to denigrate the scientific importance of “small” models.

Second, we are aware of the pitfalls and problems associated with large, integrated ABMs. The larger the model, the more care must be taken with its design, parameterization, and validation, in order to deal with the rapidly increasing levels of uncertainty [111]. Where model coupling is used, this must be done with an awareness of the scientific and technical issues involved [108, 109]. An open and transparent discussion of modeling choices and uncertainty is particularly vital where, as we advocate, model results are used to advise decision makers [36, 98].

Finally, we emphasize that agent‐based models are just one methodology among many for studying social‐ecological systems. While they have particular strengths that fit in well with certain properties of SES (e.g., heterogenous, interacting agents), they also have weaknesses that must be accounted for (e.g., the difficulty of quantitative validation) [27]. Therefore, they can only ever be one approach among several for the scientific study of SES and the providing of advice to decision makers, and must be complemented in the SES literature by studies using other empirical and theoretical methods.

## 7 Outlook

In this review, we have analyzed how agent‐based models represent social‐ecological systems in agriculture, and how such models can be made more profitable for policy advice. Looking at the current state of the field, we offer the following main recommendations for future work:

1. Develop individual‐based models of agroecosystems that are sufficiently grounded in empirical data to be useful for evaluating the impacts of policy on biodiversity and ecosystem services in specific regional contexts.
2. Integrate processes related to agricultural management practices and crop growth into individual‐based models of agroecosystems, in order to capture the spatio‐temporal dynamics of landscapes.
3. Explore the role of agricultural risk and risk management strategies in the context of climate change and environmental degradation in socio‐economic agent‐based models.
4. Use model coupling (involving agent‐based models as well as other model types) to study bidirectional interactions between the human and natural domains of agriculture, such as the possible effects of biodiversity and ecosystem services on agricultural production and farmers’ decision‐making.

By their nature, social‐ecological systems span across disciplinary boundaries. We encourage agent‐based modelers to learn to navigate the different modeling traditions that have grown up around agriculture, and to form collaborations that can help do so, in order to more holistically approach the social, economic, and environmental problems we face.

## 8 Methods

Our review methodology follows the guidelines for systematic literature reviews of agent‐based models as outlined by Achter et al. [112]. Hence, the following sections describe the processes of conceptualization, literature search and selection, and finally the structured analysis approach.

### 8.1 Conceptual Framework

To get an initial overview of the field, we interviewed two modeling experts about their experiences with agricultural social‐ecological modeling, and searched the Web of Science Core Collection using different combinations of relevant key words. The insights gained from this preliminary search provided the basis for the development of the SES concept depicted in Figure 1, as well as our search and analysis methodology.

For the purposes of this paper, we conceptualize agricultural SES as containing a human and a natural domain, each in turn containing multiple subsystems, or categories of study. In the human domain, we look at agricultural policy, farmer decision‐making, and market dynamics. In the natural domain, we include land use and land use change (i.e., landscape dynamics), and environments and ecosystems. The two domains are linked by agricultural practice, i.e., the production of crops and livestock, which is the point at which human and natural processes most directly interact. Finally, research and monitoring efforts produce information about the different parts of the system, which can help inform the actions of decision‐makers. While this concept is very simplified and glosses over many of the complexities of agricultural SES, it captures the structure of most of the models that we review, and thus reflects current research in the field.

### 8.2 Literature search and selection

For our main literature search, we then designed a search string that was intended to return sufficiently broad results while reducing the number of out‐of‐scope studies. We queried the Web of Science Core Collection with this, with the cut‐off date on the 11^th^ March 2026:

(agent‐based OR individual‐based) model AND (agric* OR farm*)

AND (policy OR market OR econom* OR “farm management” OR “agricultural practice” OR “decision making”)

AND (landscape OR “land use” OR biodiversity OR ecosystem OR environment*)

NOT (hydrology OR groundwater OR archaeology OR disease OR veterinary OR fish* OR forestry OR review)

Two authors scanned the title and abstract of all publication returned by both searches to find models that were relevant to our purpose, for which we defined four criteria:

1. The model must be at least partially an ABM/IBM.
2. It must have a direct link to agriculture.
3. It must include both human and natural processes, factors, or outcomes.
4. It must be set in the context of agroecosystems, i.e., terrestrial environments dominated by agricultural land use.

The last criterion (and the final part of the search string) excluded studies that solely focus on forests or water resources. However, we did include models that assess related topics such as deforestation or irrigation, as long as the primary focus of the model was agriculture‐related.

We tagged relevant papers by region (e.g., Europe, Asia) and environmental focus (e.g., biodiversity, nutrients, pesticides), and identified which model was used in each publication. Finally, we used a stratified sampling approach to select models for detailed analysis, considering the distribution of regional and environmental foci and preferentially selecting models with multiple publications. If a model had been used for more than one study, we additionally tried to track down its first publication as well as any recent expansions, in order to consider the full capabilities of a given model in our analysis.

### 8.3 Model Analysis

Based on the conceptual diagram of agricultural SES in Figure 1, we identified seven categories in which we compared models against each other. These include: Input Data, Policy Assessment, Economic Considerations, Agricultural Decision‐Making, Farm Production, Landscape Composition and Dynamics, and Environments and Ecosystems.

We then evaluated how models implement these categories using a total of 26 binary (yes‐or‐no) criteria (Figure 2). The criteria were developed in an iterative process, in which three of the authors suggested an initial set of criteria based on the conceptual diagram and the preliminary literature search, and then refined these in discussion with the whole author team. The aim was to develop a set of criteria that are evenly balanced among research disciplines, and can show relevant similarities and differences in the way existing model represent agricultural SES. We also wanted to look beyond established modeling approaches and include aspects that have been discussed in the wider SES literature, but are not yet commonly studied using ABMs.

The definitions for the final set of criteria are listed in Table 1. Note that while most criteria are independent of each other, the first criteria in some categories (specifically P1, E1, D1, L1, and N1) are used as “umbrella” criteria. These are meant to show whether a model considers this category at all, with the subsequent criteria in the category addressing specific modeling approaches. We used binary criteria rather than more fine‐grained approaches, as early testing showed that this improved intercoder reliability [112]. For each model, we checked which criteria it fulfils based on its description in the associated papers, and collected additional information about its scope (e.g., spatial scale, environmental outcome). The analysis was shared between three of the authors, with joint discussion whenever necessary to resolve uncertain classifications or clarify the definitions of the criteria.

## Author Contributions

**Daniel Vedder**, **Judith Josephin Rakowski**, and **Guy Pe'er** conceptualized the study. **Daniel Vedder**, **Judith Josephin Rakowski**, and **Lea‐Deborah Kolb** conducted the literature search; **Daniel Vedder**, **Lea‐Deborah Kolb**, and **Greta Sophie Theilen‐Loges** carried out the literature analysis. **Daniel Vedder** created the figures and led the writing. **Guy Pe'er** and **Sebastian Lakner** provided supervision and acquired funding. All authors co‐developed the methodology, contributed critically to drafts, and approved submission.

## Conflicts of Interest

The authors declare no conflicts of interest.

## Data Availability

The authors have nothing to report.
